# Precision medicine for respiratory diseases: A current viewpoint

**DOI:** 10.3892/mi.2024.155

**Published:** 2024-04-12

**Authors:** Vasiliki Epameinondas Georgakopoulou, Ioannis G. Lempesis, Pagona Sklapani, Nikolaos Trakas, Demetrios A. Spandidos

**Affiliations:** 1 Department of Pathophysiology, Laiko General Hospital, Medical School of National and Kapodistrian University of Athens, 11527 Athens, Greece; 2 Department of Biochemistry, Sismanogleio Hospital, 15126 Athens, Greece; 3 Laboratory of Clinical Virology, School of Medicine, University of Crete, 71003 Heraklion, Greece

**Keywords:** respiratory diseases, asthma, chronic obstructive pulmonary disease, idiopathic pulmonary fibrosis, precision medicine, lung cancer

## Abstract

In the realm of respiratory illnesses, despite the immense costs and efforts invested in diagnosis and treatment, numerous patients with chronic respiratory conditions or malignancies do not respond well to existing therapies. Delayed diagnoses and inadequate treatments contribute to these challenges, along with adverse reactions or treatment limitations due to side-effects. However, recent advancements in understanding respiratory diseases have paved the way for personalized medical treatments, considering individual genetic, molecular and environmental factors. Precision medicine, which accommodates individual differences in disease susceptibility and response to treatments, aims to improve patient care by aligning medical research with tailored therapies. Innovative technologies, such as genomic sequencing and biomarker identification contribute to this approach, allowing for customized treatments and the identification of effective therapies. Additionally, the application of precision medicine in lung cancer treatment exemplifies the forefront of individualized care within respiratory medicine. Several studies have explored the role of precision medicine in managing respiratory infectious diseases, asthma and idiopathic pulmonary fibrosis, aiming to categorize diseases more accurately and design targeted therapies. The ultimate goal is to enhance treatment effectiveness, minimize adverse events, and shift towards a patient-centered approach to managing respiratory conditions. Despite limitations, precision medicine holds promise for improving patient outcomes and emphasizing personalized care in respiratory medicine.

## Precision medicine for respiratory diseases

Respiratory illnesses pose a significant cause of illness and mortality on a global scale. Despite the substantial expenses linked to the accurate diagnosis and treatment of these conditions, a number of patients suffering from chronic respiratory diseases or malignancies cannot be effectively treated using current therapies. There are often delays in diagnoses, treatments are frequently subpar, and in several cases, patients do not receive adequate care due to treatment unavailability or poor prognoses. Additionally, some patients encounter adverse reactions or limitations in the usefulness of available treatments due to side-effects (1).

In recent years, there has been a marked improvement in the understanding of the mechanisms through which respiratory diseases develop, allowing for tailored medical treatments based on individual patient traits. Understanding the genetic roots, molecular pathways and environmental influences driving disease development has opened avenues for preventive strategies and has enhanced diagnostic and treatment strategies personalized for each patient (2).

Precision medicine, broadly defined as strategies considering individual differences in preventing and treating ailments, aims to categorize individuals into groups with varying susceptibilities, pathogeneses, manifestations and responses to therapies for specific diseases. This approach seeks to enhance patient care by combining advancements in medical research and computer science, as highlighted by the substantial allocation of funds to the Precision Medicine Initiative (3,4).

Patient stratification based on molecular and phenotypic distinctions is not a new concept in medicine. Over time, disease classifications have evolved with advances in understanding disease origins, manifestations and prognoses (5).

Cutting-edge technologies, such as whole-genome sequencing, transcriptome profiling, proteomics and imaging techniques are transforming the classification of disease subtypes. Progress in comprehending regulatory networks and essential metabolic pathways allows for adjusting immune responses and crafting specific treatments, with some already in active clinical deployment. The goal of therapeutic drug monitoring is to offer customized therapies, aiming to reduce adverse effects, while ongoing efforts focus on pinpointing biomarkers for monitoring treatment responses and refining the duration of therapies. Yet, the challenge remains in managing the vast amount of complex data, requiring integrated knowledge systems and standardized data analysis in precision medicine (6).

In due course, precision medicine is expected to lead to diagnostic tools that identify individuals who will benefit most from specific therapies, while avoiding unnecessary treatments and expenses for those who will not benefit. Sharma *et al* (7) reported the application of precision medicine in the treatment of lung cancer, an area where tailoring patient care is at the forefront within the field of respiratory medicine. They discussed significant discoveries that have advanced the current knowledge of the molecular pathways underlying the onset and spread of lung cancer, as well as the development of immune checkpoint inhibitor therapy and targeted therapies, which have significantly improved the prognosis of patients with particular subtypes of the disease (7).

Salzer *et al* (8) provided a thorough overview of the use of precision medicine in the treatment of patients with long-term respiratory infectious diseases, such as tuberculosis, nontuberculous mycobacterial pulmonary disorders and chronic pulmonary aspergillosis. They presented an overview of the use of cutting-edge therapeutic applications and diagnostics in the future, including host-directed therapies that will significantly improve patient prognoses and enable them to be cured of these diseases. They also compared the current state of care to expert management standards (8).

Heaney and McGarvey (9) evaluated the conventional method of treating respiratory conditions (asthma and chronic obstructive pulmonary disease) in stages. The impact of comorbidities on the expression of airway disease was critically examined, along with new developments in the fields of airway inflammometry, biomarkers, and companion diagnostics that characterize endotypes of severe asthma and chronic obstructive pulmonary disease and stratify the response to biologics and small-molecule inhibitors, behavioral psychology, and the introduction of quantitative tools to measure inhaler adherence using digital technologies (9).

An impartial characterization of phenotypes or endotypes (which are phenotypes classified by their mechanisms) is a crucial initial stage in advancing precision medicine for asthma. While there have been definitions and treatment targets established for allergic or eosinophilic asthma categorized as T2-high asthma, identifying non-T2 phenotypes remains a primary focus. The effectiveness of precision medicine also hinges on identifying biomarkers that can distinguish between these phenotypes, aiding in the identification of patients suitable for specific targeted therapies. Hence, precision medicine establishes connections between phenotypes (endotypes) and targeted treatments, aiming for improved outcomes (10).

Recently, Karampitsakos *et al* (11) summarized the current knowledge of precision medicine in idiopathic pulmonary fibrosis (IPF) and highlighted barriers to translating these research findings into clinical practice. Over the past decade, there has been a surge in scientific advancements, resulting in the identification of multiple biomarkers and two anti-fibrotic compounds that can attenuate the progression of IPF. The current challenge lies in translating these biomarkers into cost-effective tools that offer valuable guidance for clinicians. An essential biomarker should significantly influence decisions of clinicians, whether by aiding in early diagnosis, providing insight into disease activity, or determining the need for treatment adjustments. Achieving this could involve the use of individual biomarkers, comprehensive indexes, or polygenic risk scores (11).

Identifying biomarkers that align with disease activity could play a pivotal role in determining when intervention is necessary. Particularly for patients with interstitial lung abnormalities (ILA) and mild functional impairment, where the initiation of antifibrotic treatment is occasionally delayed, these biomarkers could prove invaluable. Additionally, biomarkers capable of identifying specific endotypes are crucial (12-14). Instead of using the term ‘idiopathic’, categorizing pulmonary fibrosis based on high-risk genomic pulmonary fibrosis, MUC5B-pulmonary fibrosis, or telomeropathy-pulmonary fibrosis may offer a more effective approach. Utilizing precision medicine and endotyping could pave the way for pharmacogenetic approaches and may help guide treatment decisions. Tailoring treatments to specific endotypes based on distinct biomarker expression levels could enhance the effectiveness of future therapies, while minimizing adverse events. Examples include ongoing clinical, such as the TELO-SCOPE Study (NCT04638517) and the study with the title ‘Low-Dose Danazol for the Treatment of Telomere Related Diseases’ (NCT03312400) testing the synthetic androgen, danazol, for patients with short telomeres, based on previous findings suggesting the potential of androgens to restore telomerase activity in IPF, as well as the PRECISIONS trial for NAC involving TOLLIP gene variants (15,16).

Transitioning from a standardized approach to a patient-centered one is crucial. Trials evaluating therapeutic biomarkers alongside the weight-based dosing of antifibrotics or investigating reduced doses in patients with ILA could offer benefits, while minimizing adverse events that often lead to treatment cessation. Encouraging studies focused on managing symptoms in a personalized manner are essential. For instance, the extended-release form of nalbuphine, acting as a dual κ opioid receptor agonist/μ opioid receptor antagonist, exhibits promise in alleviating chronic cough in patients with IPF (17-19).

**Figure 1:**
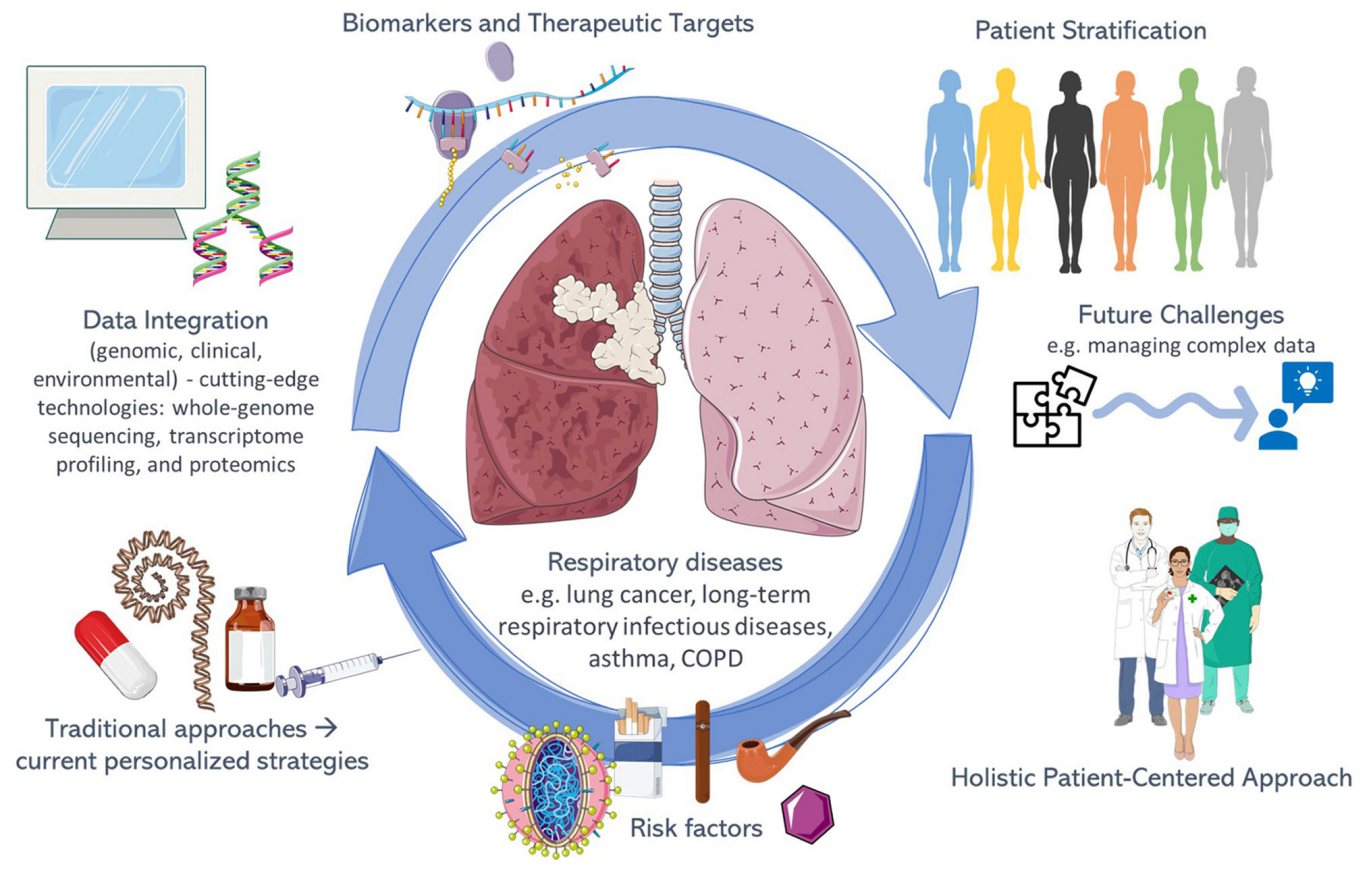
Schematic illustration representing the concept of precision medicine in the field of respiratory diseases. Parts of this image were derived from the free medical site http://smart.servier.com/ (accessed on November 15, 2023) by Servier, licenced under a Creative Commons Attribution 3.0 Unported Licence. COPD, chronic obstructive pulmonary disease.

While not all respiratory disease cases can be cured, precision medicine continues to enhance patient prognoses. It emphasizes personalized care, yet a holistic approach centered on the patient beyond scientific advancements ensures a truly individualized and humane medical practice. The concept of precision medicine in the field of respiratory diseases is illustrated in Fig. 1.

## Data Availability

Not applicable.
